# What are the difficulties in conducting randomised controlled trials of thromboprophylaxis in myeloma patients and how can we address these? Lessons from apixaban versus LMWH or aspirin as thromboprophylaxis in newly diagnosed multiple myeloma (TiMM) feasibility clinical trial

**DOI:** 10.1007/s11239-019-01891-0

**Published:** 2019-06-05

**Authors:** Zara Sayar, Julia Czuprynska, Jignesh P. Patel, Reuben Benjamin, Lara N. Roberts, Raj K. Patel, Victoria Cornelius, Roopen Arya

**Affiliations:** 1 King’s Thrombosis Centre, Department of Haematological Medicine, King’s College Hospital NHS Foundation Trust, London, SE5 9RS UK; 2 Institute of Pharmaceutical Science, King’s College London, London, UK; 3 School of Public Health, Imperial College, London, UK

**Keywords:** Multiple myeloma, DOACs, Apixaban, Venous thromboembolism, Thromboprophylaxis, Aspirin, Enoxaparin, Clinical trial

## Abstract

Routine thromboprophylaxis (TP) in newly-diagnosed multiple myeloma (NDMM) patients comprises either aspirin for standard risk patients or low molecular weight heparin for high risk patients. Studies using DOACs in cancer patients include few with myeloma. The aim of this feasibility clinical trial was to establish the foundations for creating a multicentre trial and identify any safety concerns with apixaban. Patient perspectives were sought. NDMM patients were stratified according to VTE risk and randomised to either standard TP or apixaban 2.5 mg BD and reviewed every 3 weeks throughout their chemotherapy. Two focus groups were carried out on 2 occasions at King’s College Hospital and Guy’s Hospital, London. Each lasted an hour, were recorded, transcribed and themes explored using NVivo 11. Ten patients were recruited, 2 considered high risk and received apixaban and 8 standard risk; 4 randomised to aspirin and 4 to apixaban. Five patients and 2 carers participated in the focus groups. There were no major bleeding or VTE events. Patients were not aware of the thrombotic risk associated with cancer. There is a lack of both written and verbal information on this topic. Myeloma patients were happy to be included in more than one trial simultaneously. Our study provides information on the difficulties facing physicians and patients on obtaining evidence of the safety of DOACs in the context of myeloma. Despite patients being happy to co-recruit into thromboprophylaxis trials along with chemotherapy trials this is not current practice.

EudraCT Number: 2015-002668-18

**Electronic supplementary material:**

The online version of this article (10.1007/s11239-019-01891-0) contains supplementary material, which is available to authorized users.

## Highlights

- The aims of this feasibility clinical trial were to establish the foundations for creating a multicentre trial
- Patient perspectives were sought on co-recruitment to two trials (one chemotherapy and one thromboprophylactic trial) and views on thrombotic risk in cancer
- This study suggests that apixaban should be fully evaluated for use in those with NDMM as thromboprophylaxis as there were no major bleeding or VTE events
- Patients were unaware of thrombotic risk associated with cancer
- Future myeloma thromboprophylactic trial recruitment could be improved if conducted in conjunction with chemotherapy trials, with a method of including patients already in receipt of an anticoagulant or antiplatelet agent; patients are willing to be co-recruited.

## Introduction

Myeloma patients have an increased risk of venous thromboembolism (VTE) [1] due to patient-, disease- and treatment-related factors [2]. The British Society of Haematology (BSH) suggest a risk assessment model for the prevention of VTE in myeloma patients treated with thalidomide or lenalidomide. This was stipulated in 2008 prior to the new generation of myeloma treatments that are available [3]. It suggests that for myeloma patients not receiving thalidomide or lenalidomide, thromboprophylaxis (TP) should be considered on a case-by-case basis, leaving ambiguity about thrombotic risk associated with other and newer agents. The UK Myeloma XI study demonstrated a VTE rate of 11.8% despite adequate TP according to current guidelines highlighting scope for improvement [4].

The evidence for the use of DOACs as TP in the cancer cohort is expanding. The Apixaban to Prevent Venous Thromboembolism in Patients with Cancer trial [5], aimed to assess the safety of apixaban in the context of VTE prevention in cancer. This placebo-controlled, double-blind clinical trial assessed the efficacy and safety of apixaban (2.5 mg BD) for VTE prevention in ambulatory cancer patients. The Khorana score was used to identify those with cancer at increased risk of VTE. VTE occurred in 12/288 (4.2%) patients randomised to apixaban compared with 28/275 (10.2%) patients randomised to placebo. Bleeding occurred in 10 patients (3.5%) randomised to apixaban and 5 (1.8%) to placebo. This trial included 15 myeloma patients. It concluded that apixaban is effective at reducing VTE rates in those with cancer. The CASSINI trial, using rivaroxaban 10 mg in a high-risk cancer cohort, found that during the intervention period there was a reduction in VTE in those receiving rivaroxaban (2.6% vs. 6.4%) [6]. These trials were largely conducted in cancer patients who would not usually receive ambulatory TP, unlike the myeloma cohort where the thrombotic risk is thought to be higher. The Khorana score is not used in myeloma patients.

NDMM patients often enter chemotherapy clinical trials so there is a need to understand patients’ thoughts and willingness to be enrolled into 2 trials simultaneously; one for chemotherapy and another addressing TP. The benefit of co-recruitment would allow the thrombotic risk of newer chemotherapy agents to be identified as they are being trialled so efficacy and thrombotic risk can be considered simultaneously avoiding subsequent clinical equipoise.

The PELICAN study highlighted that cancer patients are not aware of their thrombotic risk [7], despite it being the second highest cause of mortality in this cohort [8].

The aims of this feasibility clinical trial were twofold: (i) to assess the safety of apixaban as thromboprophylaxis in NDMM patients and to establish the feasibility of a multicentre trial and (ii) to establish cancer patients’ awareness of thrombosis risk, determine attitudes to being enrolled to multiple trials simultaneously and to explore patients’ views and experience of TP.

## Patients and methods

**Fig. 1:**
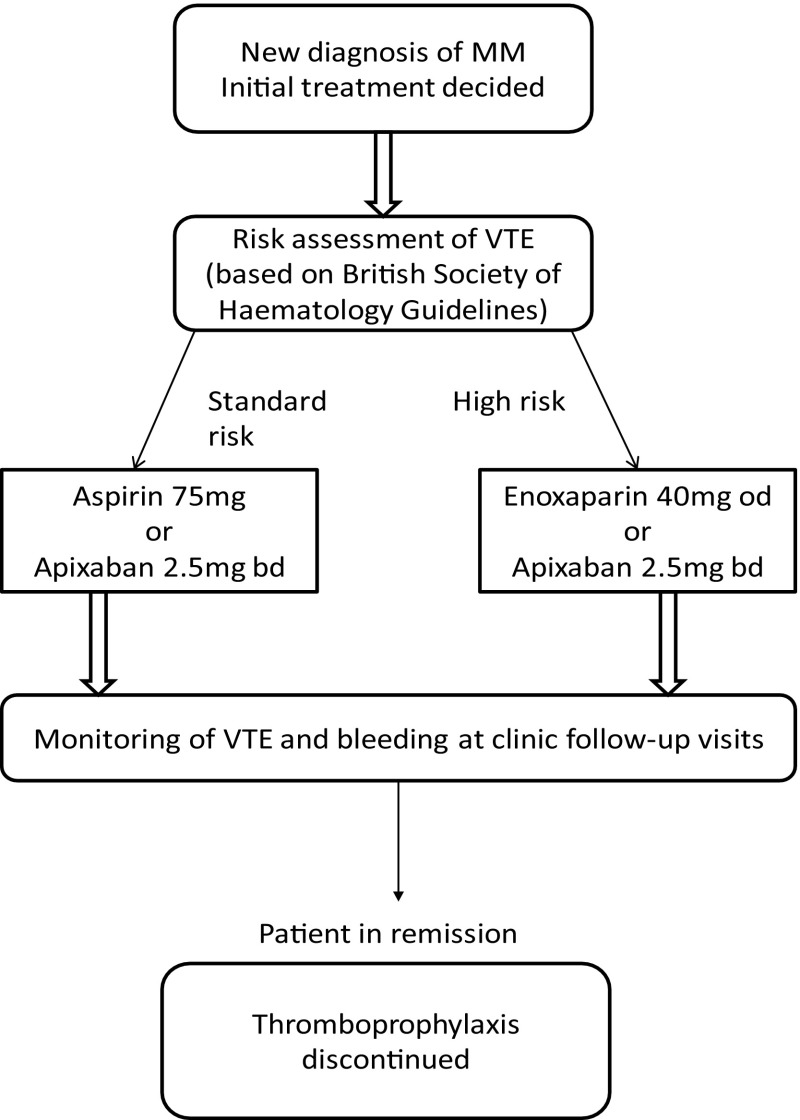
Trial protocol

King’s College Hospital NHS Foundation Trust is one of the largest teaching hospitals in the UK and includes 2 main sites; King’s College Hospital and Princess Royal University Hospital (PRUH). We conducted a randomised, open label phase IV feasibility clinical trial comparing the safety and efficacy of apixaban 2.5 mg twice a day with the standard thromboprophylactic agents (Fig. 1); enoxaparin 40 mg administered as a subcutaneous injection daily if classified as high risk of VTE, and aspirin 75 mg orally daily if considered standard risk of VTE according to the Palumbo risk assessment model [3]. NDMM patients were referred by the myeloma team, who performed the VTE risk stratification. Eligible patients were given a patient information sheet and consent was obtained from those recruited. Block randomisation was conducted following risk stratification. Patients were followed up for 6-months or until in remission.

Primary end points were bleeding requiring cessation of prophylactic therapy or an objectively diagnosed VTE.

Patients could withdraw from the study at any time for any reason. The investigator also had the right to withdraw patients from the study.

Inclusion criteria included NDMM patients requiring chemotherapy, age > 18 years and able to give written informed consent. Exclusion criteria included pregnancy or breastfeeding, established use of an anticoagulant or antiplatelet, contraindications to active substances or excipients being used and concomitant systemic treatment with strong inhibitors of both CYP3A4 and P-gp, and HIV protease inhibitors.

TP commenced on the same day as chemotherapy. Bleeding symptoms were recorded according to ISTH guidelines [9]. Patients were reviewed 1 week after starting TP to check adherence and assess for bleeding and VTE symptoms. The next visit was 3 weeks into treatment, with ongoing 3-weekly reviews coinciding with chemotherapy visits.

An electronic case report form was created and validated by the database provider (KCL CTU). This system was regulatory compliant (GCP, 21CRF11, EC Clinical Trial Directive). Source data was entered by the lead researcher (ZS).

### Focus groups

Two focus groups (FG) were conducted by the research team (ZS and JPP). The first was performed with self-selecting patients and carers who form a cancer panel in South London. They were asked about participation in trials, thoughts on the TIMM trial and knowledge, at time of diagnosis, of symptoms and risk of VTE. The second was performed with those who had participated in the TiMM trial itself to gain feedback about their experiences of the trial and co-recruitment to 2 trials simultaneously. All participants were provided with information leaflets in advance of the meetings and consent forms signed. All discussions were recorded, transcribed and analysed using NVivo 11 software. Framework analysis was used to identify predominant themes which included:

1. Knowledge of thrombotic risk
2. Concern surrounding VTE
3. Thoughts on the TiMM trial protocol, including views on co-recruitment
4. Formulation of thromboprophylactic medication

This protocol and related documents were submitted and approved by the London Central Research Ethics Committee (15/LO/131), and the Medicines and Healthcare Products Regulatory Agency (40945/0003/001-0001).

## Results

**Fig. 2:**
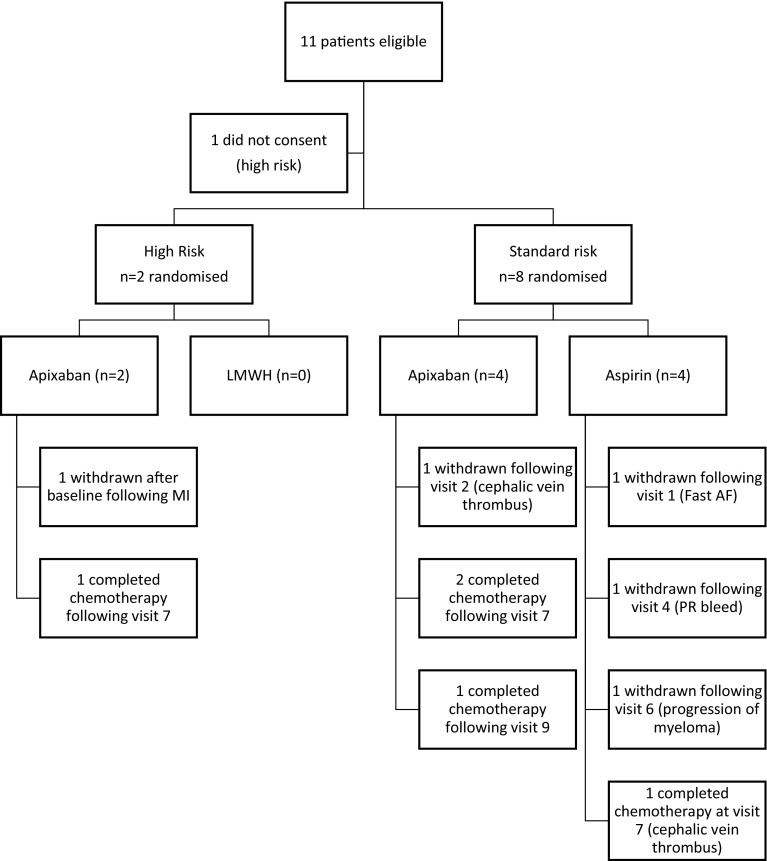
Assignment of patients and outcomes

The TiMM trial recruited from April 2016 until April 2017. Twenty-nine NDMM patients were assessed for eligibility. Eighteen patients did not meet the eligibility criteria, predominantly due to concomitant use of antiplatelets or anticoagulants (n = 11). Four patients were excluded as the CARDAMON chemotherapy trial withdrew authorisation of concurrent supportive trial participation. Ten of 11 eligible patients consented to the TiMM trial with 1 declining consent. The baseline characteristics are outlined in Table 1. Figure 2 shows assignment and outcome of patients. Three were withdrawn from the aspirin arm, and 1 from the apixaban arm.

**Table 1:** Baseline characteristics of TiMM study patients

Category	Characteristic	Aspirin	Apixaban	Total
		n = 4	n = 6	n = 10
Gender	Male sex, n (%)	2 (50)	3 (50)	5 (50)
VTE Risk	Standard	4 (100)	4 (67)	8 (80)
	High	0 (0)	2 (33)	2 (20)
Mean age, years (SD)				
		65.0 (8.6)	61.0 (10.5)	63.3 (8.6)
Ethnicity, n (%)				
	Black		2 (20)	2 (20)
	White	4 (100)	4 (80)	8 (80)
Weight kg-n (%)	< 70	1 (25)	1 (17)	2 (20)
	70 to ≤ 90	2 (50)	3 (50)	5 (50)
	> 90	1 (25)	2 (33)	3 (30)
Creatinine clearance, mL/min	≥ 80, n (%)	2 (50)	4 (67)	6 (60)
	50 to < 80, n (%)	2 (50)	2 (33)	4 (40
Previous VTE (DVT/PE)				0 (0)
Smoking status				
	Never smoker	3 (75)	6 (100)	1 (10)
	Ex-smoker	1 (25)	0 (0)	9 (90)
Myeloma treatment regimen, n (%)				
	Velcade/thalidomide/dexamethasone (VTD)	1 (25)	0 (0)	1 (10)
	Carfilzomib/cyclophosphamide/dexamethasone (CCD)	2 (50)	5 (83)	7 (70)
	Velcade/Melphalan/Prenisolone (VMP)	4 (100)	6 (100)	2 (20)
Multiple myeloma classification, n (%)				
	Stage 1	2 (50)	3 (50)	5 (50)
	Stage 2	1 (25)	3 (50)	4 (40)
	Stage 3	1 (25)	0 (0)	1 (10)
VTE risk factors, n (%)				
	Obesity (BMI > 30)	1 (25)	3 (50)	4 (40)
	Recent surgery (< 6 weeks)	0 (0)	1 (17)	1 (10)

### Efficacy

No VTE events occurred. There were 2 superficial VTE, with no extension into the deep veins, diagnosed using duplex ultrasonography during the course of the trial. Both events were associated with the use of a peripheral cannula for chemotherapy administration. One occurred on apixaban and one on aspirin; both patients were considered standard risk of VTE.

### Safety

Adverse events (AEs) included 3 bleeding events; 2 in the aspirin arm and 1 in the apixaban arm (classified as high VTE risk). All reported bleeding was clinically relevant non-major bleeding. Other AEs were considered minor and unrelated to the agents under investigation.

## Focus group results

Table 2 outlines the characteristics of those who participated.

**Table 2:** Baseline characteristics of focus group patients

Code	Age (years)	Sex (M/F)	Carer/patient?	Underlying malignancy	VTE	TP	Participated in the TiMM trial?
Focus group 1							
FG0101	69	F	Patient	Breast Cancer	Incidental PE during chemotherapy -treated with LMWH	N/A	N
FG0102	71	M	Patient	Leukaemia	N/A	N/A	N
FG0103	72	F	Carer	Husband with prostate cancer	N/A	N/A	N
FG0104	66	F	Patient	Colorectal cancer	N/A	Now has AF on warfarin	N
Focus group 2-both patients had been co-recruited to TiMM and a chemotherapy trial							
FG0201	63	M	Patient	Myeloma	No	Apixaban (high VTE risk)	Y
FG0202	60	F	Carer (Wife) of FG0201	N/A	N/A	N/A	N/A
FG0203	54	F	Patient	Myeloma	Yes-cannula associated thrombus on trial	Aspirin (low VTE risk)	Y

*VTE* venous thromboembolism, *TP* thromboprophylaxis, *N/A* not applicable, *AF* atrial fibrillation

### Knowledge of thrombotic risk

Knowledge surrounding VTE risk came from sources other than medical professionals, including family with personal experience of VTE. No panellist remembered a discussion about their VTE risk or symptoms to be vigilant of. None were given written information about this risk and struggled to find information on this topic.

I looked it up afterwards and I couldn’t find that……So I don’t think it’s greatly publicised. [FG0101].

It was widely acknowledged that at the time of receiving a diagnosis of cancer they were overwhelmed, and it may have been discussed but they volume of information received was so great that they may have forgotten.

Well, if it was, it certainly didn’t register anywhere in my brain and then I would have forgotten what was said. [FG0104].

Those who had taken part in the TiMM study were clear about their risk of VTE as it formed a large discussion with the TiMM trial team, at the time of consent.

### Concern surrounding VTE

Most felt so many changes occurred due to their cancer diagnosis that they wanted doctors to take a paternalistic approach to their treatment. They therefore do not remember feeling worried or concerned about developing a VTE.

You know the people you are consulting know a lot more about it than you do. [FG0101].

FG0101 expressed this thought initially but after developing a VTE, was more concerned about the risks associated with it which may have been related to an increase in her knowledge after the event.

### Experiences of the TiMM trial

Recruitment to the TiMM trial was viewed positively. One patient who was high risk of VTE and would have required injections was randomised to receiving oral apixaban. He viewed this favourably as he was already self-injecting insulin was relieved to avoid further injections.

Patients approved of TiMM visits coinciding with chemotherapy appointments, so no additional time or travel was required.

A positive of trial enrolment includes access to unlicensed medications. Amongst participants there was a perception that trial medications were associated with improved outcomes.

…would prefer to be treated with the newer drug - because they want to be protected. [FG0103].

By partaking in a trial, something positive could be achieved from a cancer diagnosis and the more trials they could help with, the more useful they felt.

It makes you feel good about yourself. [FG0102].

FG2 viewed their experiences of co-recruitment positively. The predominant concern about co-recruitment to trials in both FGs related to communication between teams.

Team A has been… they’ve taken several samples and later on in the day, team B comes along to take more samples……what happened to all the tubes you took this morning? Why can’t you co-ordinate? [FG0103].

The main concern patients have is related to their diagnosis of cancer. All other aspects, including VTE risk, are considered peripheral. If involved in more than one trial, their predominant concern would be related to the trial dealing directly with their diagnosis of cancer.

### Formulation of thromboprophylaxis medication

It was considered easier to omit an oral medication rather than an injectable so adherence may be improved with an injectable preparation. Taking tablets is considered a routine and common part of daily life. FG0103 suggested some patients struggling to physically inject themselves may benefit from an oral alternative.

Efficacy was the primary concern for patients rather than side effects or frequency of administration. They felt taking medications was a process that they had to go through as part of their overall treatment.

## Discussion

This feasibility clinical trial reports no safety or efficacy issues related to the use of apixaban as evidenced by no increase in VTE events or bleeding associated with apixaban use. The findings suggest patients will consent to a supportive clinical trial evaluating thromboprophylaxis whilst being treated for myeloma.

Although we did not reach our recruitment target, recruitment to the TiMM trial was good. Patients were motivated to take part; 10 of a possible 11 consented to the trial. The main issue with recruitment stemmed from the eligibility criteria. Eleven patients referred for eligibility assessment were on an alternative anticoagulant or anti-platelet agent which precluded them from the TiMM trial. When considering the design of future studies, this needs to be carefully considered and approaches which allow the inclusion of these patients would be beneficial.

Seven patients were co-recruited to TiMM and the CARDAMON study and no patient declined co-recruitment. However, due to regulatory approvals, 4 patients who would have been candidates for the TiMM study were not eligible due to already being enrolled into another study. To improve recruitment, our experience suggests future TP trials should form a supportive arm of chemotherapy trials. This would enable new chemotherapy drugs to be trialled alongside thromboprophylaxis, as would be offered in routine clinical practice, with the disadvantage of an added confounding factor.

This trial suggests apixaban and aspirin may not be sufficient to prevent cannula-associated superficial thrombophlebitis. Given that two occurred in a relatively small cohort, when chemotherapy combinations include intravenously-administered drugs, consideration needs to be given to the risk of thrombosis associated with an intravenous line and irritant chemotherapy and whether this should feature in a risk assessment is uncertain. Furthermore, it is unknown whether the advent of cannula-associated phlebitis influences subsequent VTE risk. The management of cannula-associated thrombophlebitis lacks consensus and warrants further research. The optimal duration of thromboprophylaxis remains uncertain.

Patients with cancer first and foremost see themselves as cancer patients. Their primary concerns are receiving treatment for this and everything else, including VTE prevention is secondary. This has been echoed in the PELICAN study [7]. Future VTE trials would ideally be conducted in conjunction with chemotherapy treatment. There is a lack of research on the VTE risk associated with the new therapeutic agents undergoing trials. For example, it is unclear if carfilzomib confers the same protection against VTE as other proteasome inhibitors [10]. These questions must be answered in the context of large, randomised controlled clinical trials, in conjunction with a thromboprophylaxis arm.

The efficacy and side effects of TP were the considered more important to patients than whether it was delivered orally or subcutaneously. This is supported by other work which indicates that the primary concern of cancer patients with regards to thromboprophylaxis is an agent that will cause the least interference with their cancer treatment, regardless of preparation [11].

Patients were unaware of their risk of VTE at the time of cancer diagnosis. This is likely related to being overwhelmed at diagnosis with information about their malignancy and treatment. Given that this is the second commonest cause of mortality in this cohort [8], it is important this risk is reiterated at each appointment.

## Limitations

It is undoubtedly a limitation that no high-risk patients were randomised to low molecular weight hepari (LMWH) as both were randomised to apixaban. Lower recruitment than expected impacted on the ability to assess VTE rates. Relaxing the exclusion criteria of future studies could be considered with regards to pre-existing anticoagulation or antiplatelet therapy.

Both focus groups had small numbers of patients and carers in attendance and although this allowed for a more in-depth discussion of topics, it may have been prudent to carry out more focus groups to increase numbers. The first focus group consisted of cancer patients on a cancer panel, rather than myeloma patients. They may also represent a self-selecting group of patients as they are likely to be highly motivated individuals by the nature of their presence on such a panel.

## Conclusion

This trial highlights the issues with recruiting NDMM to large randomised DOAC thromboprophylaxis trials and suggests solutions. Patients are preferentially recruited to chemotherapy trials which do not allow co-recruitment. Patients support co-recruitment to trials, or an alternative is to create a thromboprophylaxis sub-study within myeloma chemotherapy trials.

Patients often have comorbidities requiring the use of antiplatelets or anticoagulants, if patients are already on these agents, there needs to be a mechanism in trial designs by which they can be included in TP trials. VTE is still poorly understood by myeloma patients and more needs to be done to address this.

## Electronic supplementary material

Below is the link to the electronic supplementary material.

Supplementary material 1 (DOCX 18 kb)
