# Supplementary material for: What are the difficulties in conducting randomised controlled trials of thromboprophylaxis in myeloma patients and how can we address these? Lessons from apixaban versus LMWH or aspirin as thromboprophylaxis in newly diagnosed multiple myeloma (TiMM) feasibility clinical trial
Source: J Thromb Thrombolysis. 2019 Jun 5;48(2):315–22. doi: 10.1007/s11239-019-01891-0 (PMC6599493; doi:10.1007/s11239-019-01891-0)
Supplement: Supplementary file 1 — Supplementary material 1 (DOCX 18 kb) [file 11239_2019_1891_MOESM1_ESM.docx]

| **Table 3: Reasons patients did not meet the eligibility criteria for TiMM** | |
| --- | --- |
| **Reason for Exclusion** | **Number of Patients (n=)** |
| Thrombocytopenia (<50 x10^9) | 1 |
| Creatinine clearance <30ml/min | 1 |
| Already prescribed anticoagulant or antiplatelet:  *In-patient on enoxaparin*  *Out-patient on enoxaparin*  *Warfarin*  *Clopidogrel*  *Apixaban*  *Rivaroxaban*  *Aspirin* | 11  *4*  *1*  *1*  *2*  *1*  *1*  *1* |
| In another clinical trial without permission for co-recruitment | 4 |
| Unable to consent | 1 |

**Supplementary material:**

| **Table 4: Adverse events in all patients** | | | |
| --- | --- | --- | --- |
|  | **Aspirin (n)** | **Apixaban (n)** | **Total (n)** |
| **Thrombosis** |  |  |  |
| Cephalic vein thrombosis | 1 | 1 | 2 |
| **Bleeding** |  |  |  |
| PV bleeding post pessary removal | 0 | 1 | 1 |
| Mild PR bleeding | 1 | 0 | 1 |
| Bleeding post eye injection - (apixaban was omitted for 4 doses prior to this) | 0 | 1 | 1 |
| **Other Adverse Events** |  |  |  |
| One episode of temperatureᶧ | 0 | 1 | 1 |
| Myocardial infarction | 0 | 1* | 1 |
| Chromatopsia | 0 | 1 | 1 |
| Constipation | 0 | 1 | 1 |
| Light headedness | 0 | 1 | 1 |
| Abnormal liver function tests/generally unwell | 1 | 0 | 1 |
| Back ache | 1 | 0 | 1 |
| Bilateral calf tenderness | 0 | 1 | 1 |
| Diarrhoea | 0 | 1 | 1 |
| Hyperglycaemia | 0 | 1* | 1 |
| Rash | 1* | 0 | 1 |
| Red blotches | 0 | 1 | 1 |
| **Total** | **5** | **12** | **17** |
| *severe intensity events; all other events recorded as mild intensity  ᶧ As reported by patient  *PV: per vagina; PR per rectum* | | | |
